# Inhibitory effect of cervical trachea and chest wall vibrations on cough reflex sensitivity and perception of urge-to-cough in healthy male never-smokers

**DOI:** 10.1186/1745-9974-9-22

**Published:** 2013-10-02

**Authors:** Naohiro Kashiwazaki, Satoru Ebihara, Peijun Gui, Norihiro Katayama, Kumiko Ito, Ryuhei Sato, Chika Oyama, Takae Ebihara, Masahiro Kohzuki

**Affiliations:** 1 Department of Internal Medicine and Rehabilitation Science, Tohoku University Graduate School of Medicine, Seiryo-machi 1-1, Aoba-ku, Sendai,, 980–8574, Japan; 2 Department of Applied Information Sciences, Tohoku University Graduate School of Information Sciences, Aramaki Aza Aoba 6-3-09, Aoba-ku, Sendai, 980-8579, Japan; 3 Department of Geriatrics and Gerontology, Institute of Development, Aging and Cancer, Tohoku University, Seiryo-machi 4-1, Aoba-ku, Sendai, 980-8575, Japan

**Keywords:** Urge-to-cough, Dyspnea

## Abstract

**Background:**

Non-pharmacological options for symptomatic management of cough are desired. Although chest wall mechanical vibration is known to ameliorate cough reflex sensitivity, the effect of mechanical vibrations on perceptions of urge-to-cough has not been studied. Therefore, we investigated the effect of mechanical vibration of cervical trachea, chest wall and femoral muscle on cough reflex sensitivity, perceptions of urge-to-cough as well as dyspnea.

**Methods:**

Twenty-four healthy male never-smokers were investigated for cough reflex sensitivity, perceptions of the urge-to-cough and dyspnea with or without mechanical vibration. Cough reflex sensitivity and urge-to-cough were evaluated by the inhalation of citric acid. The perception of dyspnea was evaluated by Borg scores during applications of external inspiratory resistive loads. Mechanical vibration was applied by placing a vibrating tuning fork on the skin surface of cervical trachea, chest wall and femoral muscle.

**Results:**

Cervical trachea vibration significantly increased cough reflex threshold, as expressed by the lowest concentration of citric acid that elicited five or more coughs (C_5_), and urge-to-cough threshold, as expressed by the lowest concentration of citric acid that elicited urge-to-cough (C_u_), but did not significantly affect dypnea sensation during inspiratory resistive loading. On the other hand, the chest wall vibration not only significantly increased C_5_ and C_u_ but also significantly ameliorated the load-response curve of dyspnea sensation.

**Conclusions:**

Both cervical and trachea vibrations significantly inhibited cough reflex sensitivity and perception of urge-to-cough. These vibration techniques might be options for symptomatic cough management.

## Background

Cough is one of the most common medical complaints and is responsible for a significant proportion of annual ambulatory visits [1]. In up to 40% of patients with persistent cough, the cough is refractory to therapy directed at the most common causes of chronic cough [2]. The currently available medications for symptomatic management of cough are inadequate due to lack of proven efficacy and their association of undesirable or intolerable side effects at anti-tussive dosage [3], suggesting that non-pharmacological therapeutic strategies may be vital to the successful treatment of cough.

The generation of cough is regulated not only by chemical sensory inputs, the target of drug development, but also by mechanical sensory inputs [4]. Internal mechanical stimuli of respiratory tract, such as a foreign body in airway, induce cough as a defense mechanism necessary to live. In addition, external mechanical vibration of chest wall induced cough in patients with acute upper respiratory tract infection [5] and idiopathic pulmonary fibrosis [6]. External mechanical vibration of cervical trachea also induced cough in healthy adults, patients with acute upper respiratory tract infection [7,8], and respiratory patients with cough as a leading symptom [8].

Moreover, mechanical stimulation contributes not only to induction of cough by itself but also to modifying cough response generated by other reasons [9]. The cough reflex motor action thresholds to citric acid were elevated by chest wall mechanical vibration in healthy adults [10]. Intensity of reflex cough was reduced during vibrating stimulation of airway, lung and chest wall by high frequency jet ventilation in anaesthetized rabbits [11]. These studies suggest that mechanical vibration may be useful for symptomatic management of cough.

It is well established that higher centers such as the cerebral cortex or subcortical regions have an important role in both initiating and inhibiting reflexive cough [12,13]. There is general consensus that cough is typically preceded by an unpleasant awareness of an irritating stimulus and is perceived as a need to cough, termed the urge-to-cough [14]. Urge-to-cough is a component of the brain motivation system that mediates the cognitive responses of cough stimuli [15], and, theoretically, it plays pivotal role in complaints due to cough.

Heretofore, there has been no study to investigate the effects of mechanical vibration on the perceptions of urge-to-cough. Dyspnea is another unpleasant respiratory sensation, which shares several features with urge-to-cough [16,17]. There are accumulating evidences that perception of dyspnea is modulated by the chest wall mechanical vibration both in healthy subjects within an experimental setting and patients with respiratory diseases [18-21]. We hypothesized that mechanical vibration modulates not only perception of dyspnea but also urge-to-cough. Therefore, in this study, we investigated the effect of mechanical vibration of cervical trachea, chest wall and femoral muscle on cough reflex sensitivity, perceptions of both urge-to-cough and dyspnea.

## Methods

### Subjects

Twenty-four healthy male never-smokers were allocated to evaluate cough related responses to inhaled citric acid and dyspnea sensation during inspiratory resistive loads. There is well-documented desensitization of the cough reflex in smokers [22]. It is also well known that there is gender difference in the cough reflex [23]. Therefore, we focused on male healthy never-smokers in the presesent study. All were originally recruited via public postings in and around the Tohoku University School of Medicine campus. The mean age was 32.6 ±5.8 (SD) years old. The study was approved by the Institutional Review Board of the Tohoku University School of Medicine. Subjects were without history of pulmonary and airway diseases, recent (within 4 weeks) suggestive symptoms, respiratory tract infection and seasonal allergies.

### Citric acid challenge and urge-to-cough

Cough reflex challenge to citric acid was evaluated with a tidal breathing nebulized solution delivered by an ultrasonic nebulizer (NE-U17, Omron Co. Ltd., Kyoto, Japan) [17]. Details of the measurement were given elsewhere [22,23]. In brief, citric acid was dissolved in saline, providing a two-fold incremental concentration from 0.7 to 90 g/L. The duration of each citric acid inhalation was 1 min. Based on cough sound, the number of coughs was counted both audibly and visually by laboratory technicians who were unaware of the clinical details of the subjects and the study purpose [22,23]. Each subject inhaled a control solution of physiological saline followed by a progressively increasing concentration of citric acid. Each nebulizer application was separated by a 2-min interval. A control solution of physiological saline was interspersed between each change in citric acid dose. The cough reflex threshold was estimated by the lowest concentration of citric acid that elicited five or more coughs (C_5_) during 1 min.

Immediately after the completion of each nebulizer application, the subject made an estimate of the urge-to-cough on the modified Borg scale [14]. The Borg scale ranged from no need to cough (0) to maximum urge-to-cough (10). To assess the intensity of the urge-to-cough, subjects were asked to ignore other sensations, such as dyspnea, burning, irritation, choking and smoke in the throat. Subjects were told that their sensation of an urge-to-cough could increase, decrease, or stay the same during the citric acid challenges and that their use of the modified Borg scale should reflect this. We determined the initial concentration of citric acid that induced urge-to-cough sensation without provoking associated motor cough events as a threshold of urge-to-cough, termed C_u_[24].

### Perception of dyspnea

Dyspnea was induced by introducing an inspiratory resistive load to the external breathing circuit and was assessed by the modified Borg scale [22]. In brief, the sensation of dyspnea was measured while the subject breathed through a Hans-Rudolph valve with a linear inspiratory resistance (R) of 0, 10, 20 and 30 cmH_2_O/L/s. The loads were presented with increasing magnitudes. Neither ventilation nor breathing pattern was controlled during the test.

After breathing for 1 min at each level of resistance, the subject rated the sensation of dyspnea using a modified Borg scale, a category scale from which the subject selected a number from 0 (no dyspnea) to 10 (maximal dyspnea) to describe the magnitude of the sensation of dyspnea. Practically, at the beginning of the measurement, we asked each subject to rate the sensation of “kokyu-konnan” or “discomfort of breathing” while breathing with resistances. The term “kokyu-konnan” is an exact Japanese translation of “dyspnea” (“kokyu” means breathing or respiration and “konnan” means discomfort or difficulty). The term“kokyu-konnan” was not defined any further, but the subjects were instructed to avoid rating non-respiratory sensations, such as headache or irritation of the pharynx.

Immediately after scoring the Borg scale for 30 cmH_2_O/L/s-loaded breathing, the subjects was also asked to choose three phrases from a list of descriptors to best describe the quality of ongoing dyspnea. The descriptors were (1) “I feel a hunger for more air,” (2) “I feel breathless,” (3) “I cannot get enough air,” (4) “My breath does not go in all the way,” (5) “My breath does not go out all the way,” (6) “My breathing is heavy,” (7) “My breathing require force,” (8) “My breath is tiring,” (9) “My breathing requires efforts,” (10) “My chest feels tight,” (11) “My chest is constricted,” (12) “I cannot take a deep breath,” (13) “Others,” in Japanese, those are the modification of Simon et al. [25] by Nishino et al. [26]. Since it is known that there is a linear relationship between amount of load and Borg dyspnea scores [27,28], we also estimated the linear regression slope as “dyspnea slope” with least square fitting when estimated Borg scores were plotted against the corresponding amounts of resistive loads.

### Mechanical vibration

Mechanical vibration was applied by placing a vibrating tuning fork (C-128 Hz aluminum tuning fork; Niti-On Co. Ltd, Chiba, Japan) on the skin surface of each region. The tuning fork generates 128 Hz vibration of 35 dB peak amplitude with a half-life of 20 s. The tuning fork was placed on the neck over the trachea just beneath of thyroid cartridge, light intercostal muscle at the mid-clavicle, or rectus femoris at the middle thigh throughout each 1-min application of ultrasonic nebulizer and inspiratory resistive load. Since the half-life of tuning fork vibration intensity is 20 s, we continuously changed the tuning fork which we made vibrate newly every 20 s. We also placed a non-vibrating tuning fork on the cervical trachea every 20 s as a sham application.

### Experimental protocol

The subjects attended the laboratory on 4 separate days within one week. For logistical reasons, the time intervals could not be the same for all subjects. At approximately 2:00 PM in each day, the subject was evaluated for cough reflex sensitivity, and perceptions of urge-to-cough and dyspnea with one of the four applications such as cervical trachea, chest wall and femoral muscle vibrations and sham. The order of the 3 sorts of vibration (cervical trachea, chest wall, femoral muscle) and sham application was randomly selected for each subject. On the first study day, spirometry was performed according to the ATS guideline [29]. On each day, we measured cough reflex sensitivity and perception of dyspnea by cough challenge test after an evaluation of perception of dyspnea. The study protocol was approved by the local ethics committee and informed consent was obtained from all subjects.

### Data analysis

Data are expressed as mean ±standard error (SE) except where specified otherwise. The comparisons of dose–response curves were performed using two-way repeated ANOVA with Fisher’s PLSD(Protected Least Significant Difference)as a post hoc test. The mean values among the 4 groups were estimated using one-way repeated ANOVA with Fisher’s PLSD as a post hoc test. To analyze quality of dyspnea sensation during loaded, chi-square test was performed using sham application as a reference. A p value of <0.05 was considered significant.

## Results

All 24 subjects completed the experiments without any difficulty or side effects. The characteristics of subjects are summarized in Table 1. All subjects have normal lung functions.

**Table 1:** Baseline characteristics of subjects

	**N =24**
Age (years)	32.6 ± 5.8
Height (cm)	174.1 ± 6.9
Weight (kg)	71.9 ± 12.0
FEV_1_ (L)	4.22 ± 0.58
FEV_1_ (% predict)	108.0 ± 14.7
FVC (L)	4.96 ± 0.72
FVC (% predict)	118.5 ± 15.4
FEV_1_/FVC (%)	85.3 ± 0.1

Data are mean ±S.D.

Neither cervical trachea, chest wall nor femoral muscle vibrations as well as sham application induced cough by itself. However, small urge-to-cough sensations were induced by cervical trachea (0.01 ± 0.02 [SE] point), chest wall (0.09 ± 0.05 point) and femoral muscle (0.07 ± 0.03 point) vibrations and sham application (0.11 ± 0.08 point) by themselves even without tussigen inhalation. Small dyspnea sensations were induced by cervical trachea (0.11 ± 0.04 point), chest wall (0.07 ± 0.03 point) and femoral muscle (0.09 ± 0.05 point) vibrations and sham applications (0.37 ± 0.12 point) by themselves even without inspiratory resistive loads.

**Figure 1:**
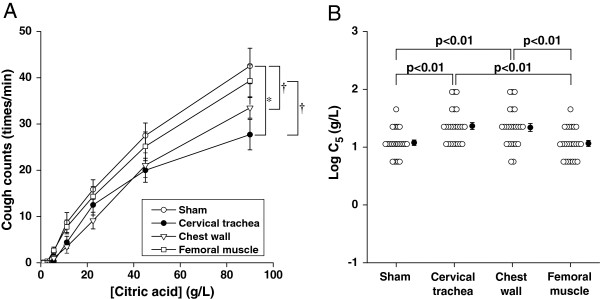
**Comparisons of cough reflex sensitivity during various external vibrations. (A)** Dose–response relationship of cough counts as a function of citric acid concentrations. Data are mean ±standard errors (SE). The comparisons among curves were performed using two-way repeated ANOVA with Fisher’s PLSD. *p<0.01, †p<0.05. The comparison between curves not indicating any symbol means no significant difference. **(B)** Cough reflex sensitivities are expressed as the log transformation of the lowest concentration of citric acid that elicited five or more coughs (C_5_). Closed circles and error bars indicate the mean value and SE in each group, respectively. P value indicated were calculated by one-way ANOVA with Fisher’s PLSD post hoc. Comparisons between the groups not indicating p value mean no significance.

Figure 1A showed the dose–response relationships of number of cough during 1-min inhalation as a function of citric acid concentration. Two-way repeated ANOVA revealed that the dose–response curve of cervical trachea and chest wall vibrations significantly differed from the sham application (p <0.01 and <0.005, respectively). The dose–response curve of cervical trachea vibration significantly differed from the femoral muscle vibration (p <0.05). When we estimated the cough reflex threshold as logC_5_ in Figure 1B, cough reflex threshold during cervical trachea (1.36 ± 0.06 [SE] g/L) was significantly greater than those during sham application (1.08 ± 0.05 g/L, p <0.01) and femoral muscle vibration (1.06 ± 0.05 g/L, p <0.01). The cough reflex threshold during chest wall vibration (1.34 ± 0.07 [SE] g/L) was significantly greater than those during sham application (p <0.01) and femoral muscle vibration (p <0.01).

**Figure 2:**
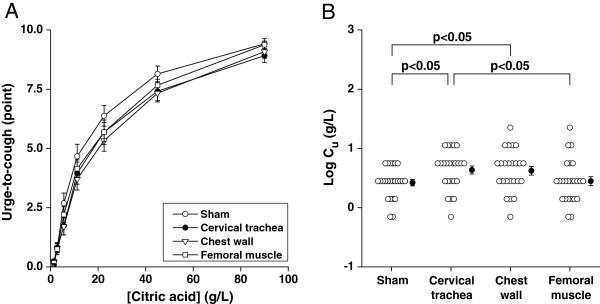
**Comparisons of urge-to-cough during various external vibrations. (A)** Dose–response relationship of urge-to-cough as a function of citric acid concentrations. Data are mean ±standard errors (SE). The comparisons among curves, calculated by two-way repeated ANOVA, failed to reveal significant difference. The comparison between curves not indicating p values means no significant difference. **(B)** Urge-to-cough sensitivities are expressed as the log transformation of the lowest concentration of citric acid that elicited urge-to-cough (C_u_). Closed circles and error bars indicate the mean value and SE in each group, respectively. P values were calculated by one-way ANOVA with Fisher’s PLSD post hoc. Comparisons between the groups not indicating p value mean no significant difference.

Figure 2A showed the dose–response relationships of urge-to-cough during 1-min inhalation as a function of citric acid concentration. Two-way repeated ANOVA revealed that there was no significant difference among 4 dose–response curves. When we estimated the urge-to-cough threshold as logC_u_ in Figure 2B, the threshold during cervical trachea vibration (0.64 ± 0.07 [SE] g/L) was significantly greater than that during sham application (0.42 ± 0.05 g/L) and femoral muscle (0.45 ± 0.07 g/L) vibrations. The urge-to-cough threshold during chest wall vibration (0.62 ± 0.07 g/L) was significantly greater than sham application (p <0.05) whereas it did not significantly differ from that during femoral muscle vibration.

**Figure 3:**
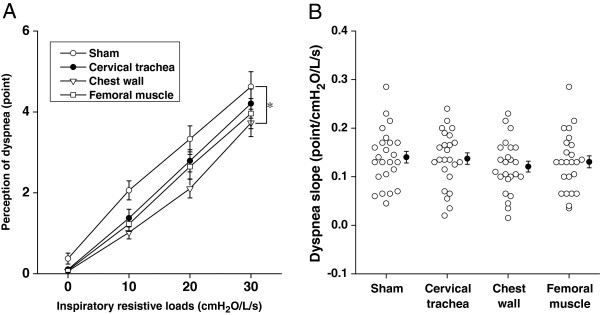
**Comparisons of dyspnea during various external vibrations. (A)** Dyspnea sensations were shown as a function of inspiratory resistive loads imposed externally. Data are mean ±standard errors (SE). The p values among curves were calculated by two-way repeated ANOVA with Fisher’s PLSD post hoc. *p<0.01. The comparison between curves not indicating any symbol means no significant difference. **(B)** Dyspnea slope calculated by the linear regression when dyspnea Borg scores were plotted as a function of resistive loads. Closed circles and error bars indicate the mean value and SE in each group, respectively. P values were calculated by one-way ANOVA with Fisher’s PLSD post hoc. Comparisons between the groups not indicating p value mean no significant difference.

Figure 3A shows the Borg scores of dyspnea as a function of inspiratory resistive loads imposed for 1-min. Two-way repeated ANOVA revealed that there was a significant difference between load-response curves for sham and chest wall vibration (p<0.05). When we estimated the slope of dyspnea response by linear regression of loads and Borg scores, there was no significant difference among slopes of sham application (0.14 ± 0.01 [SE] point/cmH_2_O/L/s), cervical trachea (0.14 ± 0.01 point/cmH_2_O/L/s), chest wall (0.12 ± 0.01 point/cmH_2_O/L/s) and femoral muscle (0.13 ± 0.01 point/cmH_2_O/L/s) vibrations (Figure 3B).

**Figure 4:**
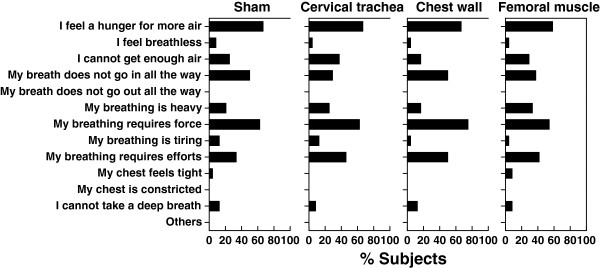
**Quantitative estimation in dyspnea induced by inspiratory respiratory load during different vibrations.** No significant difference was observed in each vibration reference to sham application by chi-square test.

Figure 4 shows the descriptors selected by the subjects. Since there was no significant difference between sham and other stimulations, the selected descriptors in each stimulation were quite comparable with sham application, suggesting that the vibrations may alter quantity but not quality of dyspnea during inspiratory resistive-loaded breathing in each subjects.

## Discussion

In this study, we demonstrated that cervical trachea vibration significantly inhibited cough reflex sensitivity and perception of urge-to-cough induced by citric acid inhalation but did not significantly affect dyspnea during inspiratory resistive load. On the other hand, the chest wall vibration significantly inhibited cough reflex sensitivity and perception of urge-to-cough as well as perception of dyspnea.

There have been two studies describing that the external vibration of cervical trachea itself induced cough. Using modified shaver to vibrate, Lee and Eccles induced cough in patients with acute upper respiratory tract infection and, much lesser extent, in healthy subjects [7]. Using tuning fork, Kamimura et al. induced cough and itchy sensation in respectively 37% and 50% of respiratory patients with cough as a leading symptoms such as acute upper respiratory tract infection, bronchial asthma, cough variant asthma and acute bronchitis [8]. In the present study, tracheal vibration by tuning fork failed to induce cough in healthy adults, suggesting that the capability to induce cough depends on the degree of inflammation and the method of vibration such as intensity, duration and frequency.

Heretofore, the effect of cervical trachea vibration on cough response experimentally induced by tussive agents has not been investigated. Kondo et al. demonstrated that the cough reflex sensitivity was inhibited by chest wall vibration in healthy adults [10], which was consistent with our result (Figure 1). In the present study, we showed that both cervical trachea and chest wall vibrations inhibited not only cough reflex sensitivity but also the perception of urge-to-cough, which is unpleasant respiratory sensation and is associated with cough-related quality of life [30], suggesting the possible benefit of symptomatic control in cough. It is well established that mechanical vibration of chest wall, especially during inspiration phase, reduces dyspnea in both experimental and pathological conditions [18,19], which is comparable to the present results (Figure 3). A randomized controlled clinical trial showed that high frequency chest wall oscillation which applied through a pneumatic vest worn over the thorax improved dyspnea in patients with chronic obstructive pulmonary disease [20]. The mechanism by which chest wall vibration improves dyspnea is thought to be attributed to the increased afferent information from the intercostal muscle spindles, which easily respond to vibratory stimulation [21]. This is consistent with our result where magnitude of dyspnea was not significantly inhibited by cervical trachea vibration.

It is certain that the vibration stimulated cutaneous mechanoreceptors. It is also likely that the cutaneous stimulation was competing for cognitive resources with dyspnea and cough sensations, resulting in inhibitory effect on cough reflex. However, the femoral muscle vibration, which also stimulated cutaneous mechanoreceptor, had no effect in the present study, suggesting the contribution of respiratory-related mechanoreceptor rather than general cutaneous mechanoreceptor. Similarly, the effect of distraction was also unlikely because the sham application had no significant influence.

The perceptual sensitivity including dyspnea sensitivity for magnitude estimation of the load stimuli follows the Stevens’ power law [31,32]. In this study, however, the dyspnea slope was estimated by the simple linear regression between magnitude of resistive loads and Borg scores without log-transformations due to the limited variations of resistive loads. Therefore, it is a study limitation that the dyspnea slope was not estimated by the psychophysiological measure of perceptual sensitivity defined by Stevens [33].

Dyspnea is a complicated sensation that recognizes several different descriptors [25]. Therefore, we attempted to comprehensively evaluate which of these descriptors are mostly affected. In this study, the choice of descriptors during loaded breathing was consistent with a previous report [26] and not affected by the vibrations. However, since quality of dyspnea differ depends on the way of loading and inspiratory resistive loading may not properly reflect the natural condition of dyspnea, further studies using different types of respiratory loadings were warranted.

Respiratory sensations such as various types of dyspnea and an urge-to-cough are the result of sensory activation of subcortical and cortical neural pathways. The brain imaging studies showed some of the cortical areas are shared across respiratory modalities while activations of some cortical areas are modality specific [34,35]. Although it is still not clear how these brain regions relate to the respiratory sensations, our study may suggest that the cervical trachea and chest wall vibrations may suppress the activation of urge-to-cough-specific and dyspnea-specific cortical areas, respectively, but not common areas for the two respiratory modalities. Further studies including brain imaging studies are necessary to elucidate the mechanism how vibrations inhibited cough reflex and urge-to-cough.

Chest wall vibration is frequently used as physiotherapy in patients complaining of sputum production and ~100 Hz is commonly used in clinical practice. Here, we found that not only chest wall but also cervical trachea vibration inhibited both cough reflex sensitivity and perception of urge-to-cough. Since the effects of these vibration techniques were weak, we should consider combining the physiotherapy with antitussive medications in order to alleviate the symptomatic cough. Further studies in patients are warranted to translate the present results in clinical setting.

## Conclusions

Our study demonstrated that cervical trachea vibration significantly inhibited cough reflex sensitivity and perception of urge-to-cough but did not significantly affect dyspnea. The chest wall vibration significantly inhibited cough reflex sensitivity and perception of urge-to-cough as well as dyspnea. Both cervical trachea and chest wall vibrations might be option for symptomatic cough management.

## Abbreviations

C5: The lowest concentration of citric acid that elicited five or more coughs; Cu: The lowest concentration of citric acid that elicited urge-to-cough.

## Competing interests

The authors declare that they have no competing interests.

## Authors’ contributions

SE participated in the design of the study, collected and analyzed data, and drafted the manuscript. NK, PG, KI, RS and CO participated in the design of the study and collected the data. TE and MK participated in design of the study and helped to draft the manuscript. All the authors read and approved the final manuscript.
